# Validation of the Spanish Version of the Lucidity and Consciousness in Dreams Scale

**DOI:** 10.3389/fpsyg.2021.742438

**Published:** 2021-10-21

**Authors:** Javier García-Campayo, Nieves Moyano, Marta Modrego-Alarcón, Paola Herrera-Mercadal, Marta Puebla-Guedea, Daniel Campos, Santiago Gascón

**Affiliations:** ^1^Department of Medicine, Psychiatry and Dermatology, University of Zaragoza, Zaragoza, Spain; ^2^Primary Care Prevention and Health Promotion Research Network, Madrid, Spain; ^3^Hospital Universitario Miguel Servet, Zaragoza, Spain; ^4^Instituto de Investigación Sanitaria de Aragón (IIS Aragón), Zaragoza, Spain; ^5^Department of Psychology, Faculty of Humanities and Educational Sciences, University of Jaén, Jaén, Spain; ^6^Department of Psychology and Sociology, Faculty of Humanities and Educational Sciences, University of Zaragoza, Huesca, Spain; ^7^Department of Psychology and Sociology, Faculty of Social and Labor Sciences, University of Zaragoza, Zaragoza, Spain; ^8^Department of Psychology and Sociology, Faculty of Social Sciences and Humanities, University of Zaragoza, Teruel, Spain

**Keywords:** consciousness, lucidity, dreams, insight, meditation, mindfulness, emotion

## Abstract

Lucid dreaming, a specific phenomenon of dream consciousness, refers to the experience being aware that one is dreaming. The primary aim of this research was to validate a Spanish version of the Lucidity and Consciousness in Dreams scale (LuCiD). A secondary aim was to explore whether meditation experience and mindfulness trait were related to LuCiD scores. Data from 367 Spanish men (34.6%) and women (65.4%) who completed LuCiD, the Five Facets of Mindfulness Questionnaire (FFMQ), and the Positive and Negative Affect Schedule (PANAS) were examined. From the total sample, 40.3% indicated some experience with formal meditation (meditators), while 59.7% did not have any meditation experience (non-meditators). A random subsample of 101 participants, who completed LuCiD for a second time after a period of 10–15days, was used for test–retest reliability analysis. The LuCiD scale comprises 28 items distributed across eight factors: insight, control, thought, realism, memory, dissociation, negative emotion, and positive emotion. Factor structure, reliability by both internal consistency and test–retest reliability, and construct and concurrent validity were tested. Confirmatory factor analysis (CFA) confirmed the original eight-factor model, showing goodness of fit in contrast to a single-factor model. Item 15 was deleted from the *Dissociation* factor as it performed poorly (i.e., skewness and kurtosis, non-normal distribution of responses, and corrected item–total correlation under 0.40). The scale showed adequate values of internal consistency (between *α*=0.65 for *Memory* and *α*=0.83 for *Positive Emotion*) and test–retest reliability by significant Pearson correlations (*p*<0.001) for each factor. The scores of meditators were higher for the LuCiD scale *Insight* and *Dissociation* factors, in contrast to those of non-meditators. The *Observing* facet of mindfulness was positively associated with all LuCiD factors, except *Realism* and *Positive Emotion*, and the *Acting with Awareness* facet showed a negative correlation with the LuCiD factor *Realism*. Finally, positive and negative affects was associated with the LuCiD factors *Positive Emotion* and *Negative Emotion*. This study provides a valid and reliable measure for exploring lucidity and consciousness in dreams for a Spanish population, Moreover, the results suggest a relationship with meditation experience, mindfulness trait, and positive and negative affect.

## Introduction

Lucid dreaming is a specific phenomenon of dream consciousness that has been defined as the experience of knowing one is dreaming while one is dreaming (LaBerge, 1985, 2009). This has been described throughout human history and is a field of growing scientific interest (Van Eeden, 1913; LaBerge, 1990; Harb et al., 2016; Saunders et al., 2016; Aspy, 2020; Yu and Shen, 2020; Ferreira et al., 2021).

Recent research has pointed out that 55% of adults have experienced at least one lucid dream (LD) and 23% experience LDs regularly (once per month or more; Saunders et al., 2016). Lucid dreaming occurs naturally during brain maturation; it appears spontaneously in the course of adolescence, and its frequency is reduced at the age of 16, although it is also susceptible to auto-suggestion and training (Stumbrys et al., 2012; Voss et al., 2012).

It has been suggested that deliberate control is possible in approximately one-third of LDs (Soffer-Dudek, 2020; for example, changing location and deliberately waking up; Aspy, 2020), although it seems to be dependent on specific aspects of control (e.g., whether controlling the dream body or the environment) where higher rates were found (Stumbrys and Erlacher, 2017). Stumbrys and Erlacher (2017) pointed out that full control over the dream plot is possible in about two-thirds of cases, while control of the dream environment and the ability to maintain dream awareness are possible in less than half of cases, where the main predictors of LD control were higher LD frequency and dispositional mindfulness in wakefulness, as well as younger age.

The frequency of LDs in adults may be related to knowing about the phenomenon or having heard about it, and according to Neuhäusler et al. (2018), an increase in age is negatively correlated with previous knowledge of lucid dreaming, whereas female gender and higher education show a positive correlation with knowledge about lucid dreaming *via* literature and friends. Another study found that lucid dreamers were socially bold, dominant, experimenting, enthusiastic, and warm (Gruber et al., 1995). Individual differences in lucid dreaming frequency are large, and authors have also related it to openness to experiences, agreeableness, and neuroticism (although the effect of lucid dreaming frequency was no longer significant when controlled for nightmare frequency; Hess et al., 2017). According to Gasca and García-Campayo (2017), the frequency of LDs has been also associated with the locus of internal control, field independence (i.e., isolating relevant elements from context, without being distracted by the context in which these elements are found), openness to experience, increased creativity, and need for cognition (i.e., motivation and preference toward the activity of thinking).

While spontaneous LDs have been reported, their deliberate induction has also become widespread (Soffer-Dudek, 2020), and several techniques have been proposed and tested (e.g., reality testing, mnemonic induction of lucid dreams technique, and incubation of external stimuli into dreams; Stumbrys et al., 2012; Mota-Rolim et al., 2019; Aspy, 2020).

Research on LDs has shown its potential benefits and applications (e.g., Schädlich and Erlacher, 2012; Stumbrys et al., 2016). Lucid dreaming has been linked to mental health and well-being, increased self-confidence, psychological resilience, and positive emotions (Soffer-Dudek, 2020). Using an online survey, Schädlich and Erlacher (2012) reported five main reasons for which people used LDs: simply having fun (approximately 80%; e.g., flying, playing games, dancing, etc.); they benefit from lucid dreams by decreasing their nightmare frequency and intensity (approximately 60%); other applications such as problem-solving (approximately 30%), development of creativity (approximately 27%), and practice of specific movements (approximately 21%).

As a therapeutic approach, lucid dream therapy (LDT), i.e., training patients in induction techniques, has shown utility in the treatment of nightmares (e.g., Lancee et al., 2010; Holzinger et al., 2015; Macêdo et al., 2019), motor skills practice (Erlacher and Schredl, 2010; Schädlich et al., 2017), and treatment of traumatic stress (Soffer-Dudek et al., 2011). For the treatment of post-traumatic stress disorder (PTSD), although preliminary evidence was found for military veterans (Harb et al., 2016), LDT has not shown any beneficial effects for PTSD symptoms (Soffer-Dudek, 2020).

Despite the potential benefits and therapeutic applications of lucid dreaming, to date, the effects reported in most studies have been weak and inconsistent, and the mechanism of chance remains unclear – where gaining control, rather than dream awareness *per se*, may be responsible for the improvement – with no evidence supporting LDT over other evidence-based therapies (Lancee et al., 2010). For these reasons, Soffer-Dudek (2020) concluded that more research is needed on the applications of lucid dreaming, the adverse consequences of LD induction (e.g., sleep quality and psychological reality – fantasy boundaries have scarcely been investigated), and whether training people to achieve LDs is worthwhile. In this regard, Aviram and Soffer-Dudek (2018) also suggested that lucidity should not be considered necessarily suggestive of well-being. LDs may be positive or negative, depending on lucidity characteristics. These authors developed an expansive measure of several LD characteristics (the Frequency and Intensity Lucid Dream questionnaire; FILD) and explored their relations with symptomatology. In short, they concluded that lucidity characterized by high intensity (particularly, control activity and certainty of dreaming) and positive affect is related to fewer psychopathological tendencies.

The increased popularity of and scientific interest in LDs in recent years may be due in part to advances in their theoretical background and conceptualization. There is a consensus in describing LD as a continuum with different degrees of consciousness rather than a dichotomic experience (e.g., Barrett, 1992; Stumbrys et al., 2012; Gasca and García-Campayo, 2017). In this regard, Voss et al. (2013) defined lucid dreaming as hybrid states of consciousness in which part of the brain operates in the primary mode, while another part has access to secondary consciousness (i.e., the dreamer is aware of the fact that he/she is dreaming while the dream continues). Independent laboratories have validated the existence of LDs by identifying their neurophysiological correlates and showing distinct patterns of brain activation that supports the hypothesis of hybrid states with elements of primary and secondary consciousness modes (e.g., Voss et al., 2009, 2014; Dresler et al., 2012).

According to hypothesis of Hobson and Voss (2010, 2011), based on assumptions of Edelman (2003, 2005), the delusional character of ordinary dreams is caused by a predominance of the primary mode of consciousness, a distinct space in the consciousness continuum (i.e., lower-level consciousness) that is deprived of the ability to control, and characterized by a fusion of past, present, and future, where perception and emotion predominate. In contrast, the second secondary mode (higher-order consciousness), characterized by self-reflection and metacognition, enables planning ahead, reflecting on the past and contemplating the future (Hobson, 2009). Several studies have found that cognitive processing during wakefulness and sleep are related (Gasca and García-Campayo, 2017).

To measure and assess major and minor determinants of LDs, Voss et al. (2013) developed the Lucidity and Consciousness in Dreams scale (LuCiD) based on theoretical considerations and empirical observations. The authors identified eight factors, primarily based on the work by LaBerge and DeGracia (2000) and Kahn (2007), from an exploratory analysis that was validated using confirmatory analysis. These factors were proposed to differentiate between lower (primary) and higher (secondary) level consciousness in dreams, as well as to identify lucid vs. non-lucid dreams. Thus, the authors were interested in exploring the phenomenological correlates of primary and secondary consciousness in dreams (lucid and non-lucid). An initial formulation of 50 items was made by an interdisciplinary team of philosophers, psychiatrists, and psychologists and based on reports from lucid dreamers and theoretical considerations. It was subsequently reduced to 24 items in a second version before the addition of four new items regarding positive and negative emotion, specifically. The final validated LuCiD comprises 28 items distributed across eight factors involved in LD consciousness that can best be described by them (Voss et al., 2013; Voss and Hobson, 2015): (1) lucid insight (Insight; *α*=0.91) into the fact that what one is currently experiencing is not real but only a dream; (2) control over thought and actions in dreams (i.e., control over dream plot; Control; *α*=0.90); (3) logical thought about other dream characters (Thought; *α*=0.82), (4) perceptual realism (Realism; *α*=0.79) pertaining to the similarity between emotions, thoughts and events, with wakefulness as judged after awakening from the dream; (5) memory access to elements of waking life (Memory; *α*=0.66); (6) experiencing the dream from a third person perspective (Dissociation; *α*=0.56); (7) negative emotion (Negative emotion; *α*=0.68); and (8) positive emotion (Positive emotion; *α*=0.87). The eight-factor model was supported by exploratory and confirmatory factorial analyses where the leading factor was insight, followed by thought, control, positive emotion, and dissociation. The results showed that the factors that mainly distinguish LDs from ordinary dreams are both insight and control. Moreover, when comparing the scores of each factor for lucid vs. non-lucid dreams, all factors are shown to be involved in dream consciousness, except for realism and negative emotion, which do not differentiate between lucid and non-lucid dreams. This suggests that lucid insight is separable from both bizarreness in dreams and a change in the subjectively experienced realism of the dream. According to Voss and Hobson (2015), it is interesting that the factor analysis result supported both the restricted and broader definition of the LD. In particular, the strength obtained from the insight favors the simple definition, while the wide range of other factors favors the more complex definition. However, formal comparisons using factorial analysis for an eight- and single-factor global model (i.e., the use of one global factor to explain lucidity and consciousness in dreams) were not tested by the authors.

In summary, the results from Voss et al. (2013) showed LuCiD to be a reliable measure for assessing and quantifying lucidity and consciousness in dreams as a subjective experience, suggesting that secondary consciousness adds cognitive functions and positive emotionality to primary consciousness. Therefore, the development and validation of LuCiD facilitate understanding of the phenomenology o LDs and present an opportunity to further investigate LDs in different populations, cultures, as well as their relationship to other psychological constructs. Nevertheless, as far as we are aware, data compiled from the use of LuCiD are scarce, and validation in other languages is only available from the original version.

As previously mentioned, lucid dreaming may cover a variety of applications for the dreamer himself or herself and indeed has been proposed as a promising tool for obtaining deeper insight into the mind–body relationship (e.g., Neuhäusler et al., 2018). Thus, exploring the relationship between lucid dreaming and mind–body practices such as mindfulness – the awareness that arises through paying attention, on purpose, in the present moment, non-judgmentally (Kabat-Zinn, 2013) – may help us to better understand awareness and the relationship between awake and dream states. Gasca and García-Campayo (2017) suggest that mindfulness in waking states is related to LDs, based on the evidence concluding that mindfulness practice promotes metacognitive skills. Yu and Shen (2020) found that individuals with higher metacognition traits would have more metacognition activities in dreams and lower dream bizarreness values. Therefore, according to Gasca and García-Campayo (2017), metacognition may be a key mechanism of action meditating mindfulness practice and lucidity in dreams. Several studies have found preliminary evidence supporting the relation between mindfulness (trait), meditation experience, and frequency of LDs (e.g., Hunt and Ogilvie, 1988; Stumbrys et al., 2015; Baird et al., 2019). Nevertheless, the literature is scarce as regards mindfulness, meditation, and lucid dreaming.

The primary aim of this research was to validate a Spanish version of LuCiD. A secondary aim was to explore whether meditation experience and mindfulness trait were related to LuCiD scores.

The following hypotheses were tested:

H1: We expected to provide structural validity for the eight-factor model previously proposed by Voss et al. (2013), with adequate reliability (internal consistency and test–retest). Two models were compared: (a) one derived from the original proposal (Voss et al., 2013), composed of a set of eight factors and (b) a single-factor model in which items only belong to one global factor.

H2: As evidence for validity, we examined the relationship between LuCiD scores and the following sociodemographic variables: sex, age, and other meditation-related variables, such as whether subjects practice meditation and have experience with meditation and how long they have been meditating for. Moreover, the relationship between LuCiD scores and other measures, such as the facets of mindfulness and positive or negative affect, will be examined. In particular, we expect to find significant associations between LuCiD scores and meditation experience and mindfulness facets.

## Materials and Methods

### Participants

Sample size calculation was carried out following the rule of thumb 10:1 ratio (participants×items) to estimate the minimum number of participants needed, taking into consideration the number of variables (Kline, 2011; *N*=280). Considering the 20% missing data rate, a minimum final sample of 336 participants was required.

Data from 579 recruited men and women were used. Data from 149 individuals were eliminated because they indicated nationalities other than Spanish. We also considered depressive or anxiety disorder as exclusion criterion. Fifty-eight participants indicated having anxiety disorder, 14 of them also indicated having a depressive disorder, and five only reported a depression disorder. Therefore, data from 63 cases were discarded. The final sample was made up of 367 data points (34.6% men, 65.4% women) from the general population. They were all of Spanish nationality, and their age range was 17–73years (*M*=43.12; *SD*=11.86). Most of the sample was married or had common-law partners (51.8%), or single (38.7%), with 7.4% separated or divorced, and 2.2% widowed. Based on the highest level of education completed, 75.2% of the sample had a university degree. Approximately 40.3% of the sample indicated having experience with formal meditation (meditators; *N*=148), while 59.7% did not (non-meditators; *N*=219). Participants with meditation experience reported that they meditated a mean (M) of 24.28min (*SD*=14.01) whenever they practiced formal meditation. Length of participants’ experience was as follows: less than 1year (15.3%), between 1 and 3years (31.9%), between 4 and 6years (24.3%), between 7 and 9years (11.1%), and more than 10years (17.4%). They had interrupted their formal practice during their lives for a mean of 7.86months (*SD*=8.78) ranging from 0 to 50. The following percentages of time dedicated to each type of meditation practice were reported: concentrative or focused attention (*M*=48.31; *SD*=28.45), open monitoring (*M*=23.53; *SD*=23.32), Compassion/Loving-Kindness (*M*=17.45; *SD*=23.1), values (*M*=14.15; *SD*=23.21), deconstructive/non-duality (*M*=7.85; SD=16.32), and informal practice (*M*=25.45; *SD*=27.08).

Of the total sample, 101 participants completed LuCiD twice, of whom 32.7% were men and 67.3% were women.

### Procedures

First, translation and adaptation of the LuCiD items to Spanish were carried out individually by two researchers who were unaware of the objectives of the questionnaire. In this step, experts in LD were asked about the adequacy and consistency of the translated items. Second, an English-speaking and bilingual linguistic expert with no specific knowledge regarding the instrument performed back-translations. Any differences between the translations were resolved through mutual agreement. Both translators and authors were present during the agreement. The authors were familiar with written technical English, and the psychological construct assessed using the questionnaire. The usual guidelines were followed for cross-cultural adaptations (e.g., Guillemin et al., 1993). As all the items achieved 85% agreement regarding their clarity, no changes were made in this phase. The Spanish version of LuCiD can be seen in Supplementary Material.

The final version was built up into an online version.^1^ The survey containing the questionnaires was disseminated through several different social media (e.g., Twitter, Facebook) and university notices (specifically, as part of the Master in Mindfulness Programme at the University of Zaragoza). The link was also posted on a LD website.^2^ When participants accessed the link, an informed consent form containing the main goal of the study and the name of the study leader was provided. Only participants who gave their informed consent by checking a box confirming their willingness to take part in the study were able to access the survey. The estimated time to complete the questionnaire was 25min. A number of participants (a random subsample) had to complete the questionnaire a second time (after 10–15weeks) to provide test–retest reliability. For those cases, each participant had to indicate his/her initials and his/her consent to be contacted in order to perform the retest (email contact). This served as the code with which to match their answers collected for the second time. No other identification system was required from the participants, assuring their anonymity. This study was approved by the Clinical Research Ethics Committee of Aragón (Comité Ético de Investigación Clínica de Aragón, CEICANo. 12/2020; 27 May, 2020) which is part of the Health Research Institute of Aragon (Instituto de Investigación Sanitaria de Aragón, IIS Aragón), Zaragoza, Spain. This committee is responsible for evaluating all research projects involving people or personal data from the University of Zaragoza.

### Measures

#### Sociodemographic Questionnaire

The participants were asked about their gender, age (in years), nationality, current city of residence, marital status (single, married, cohabiting couple, separated/divorced, widowed, and other), education (graduate/bachelor’s degree/diploma, master’s degree, doctorate, and others), and some information regarding their experience with meditation, such as whether they had meditation experience (“yes” or “no”), the mean in minutes for each time that they practiced formal meditation, length of meditation experience (i.e., less than 1year, between 1 and 3years, between 4 and 6years, between 7 and 9years, or more than 10years), the time (in months) that they may have interrupted their formal practice during their lives, and the percentage of their time dedicated to each type of formal meditation (concentrative or focused attention, open monitoring, Compassion/Loving-Kindness, values, deconstructive/non-duality, and informal practice). A brief explanation of each type of meditation practice was provided. The meditation information provided and collected was based on author recommendations and previous studies (e.g., Soler et al., 2014; Dahl et al., 2015; Campos et al., 2019).

#### Lucidity and Consciousness in Dreams Scale

Lucidity and consciousness in dreams scale is a self-reported measure to assess different aspects of lucidity and consciousness in dreams (Voss et al. 2013). It comprises 28 items distributed across eight factors: Insight (i.e., *while dreaming, I was aware of the fact that the things I was experiencing in the dream were not real*), Control (i.e., *in my dream, I was able to manipulate or control other dream characters in a way that would be impossible in waking*), Thought (i.e., *while dreaming, I often thought about my own actions*), Realism (i.e., *the emotions I experienced in my dream were exactly the same as those I would experience in such a situation during wakefulness*), Memory (i.e., *while dreaming, I was able to remember my intention to do certain things in the dream*), Dissociation (i.e., *while dreaming, I saw myself from outside*), Negative Emotion (i.e., *while dreaming, I had strong negative feelings*), and Positive Emotion (i.e., *while dreaming, I had strong positive feelings*). The items are answered on a six-point Likert scale that ranges between 0=strongly disagree to 5=strongly agree. Participants were instructed to select a recent dream that he/she had in order to answer the corresponding questions. Evidence of construct validity was provided by exploratory and confirmatory factorial analyses that supported the eight-factor models. Cronbach’s alpha reliability values for the subscales were good for most factors, as shown above, which the authors attributed to the heterogeneity of the constructs.

#### 15-Item Five Facet Mindfulness Questionnaire

15-item five facet mindfulness questionnaire (FFMQ-15; Feliu-Soler et al., 2021) is a 15-item short-form version of the Spanish-validated FFMQ (Cebolla et al., 2012) that measures trait-like tendency to be mindful in daily life and comprises five different facets of mindfulness: (i) *observing*, which refers to the individual’s capacity to pay attention to internal and external experiences such as sensations, thoughts, and emotions; (ii) *describing*, which assesses the ability to describe events and personal responses in words; (iii) *acting with awareness*, which involves focusing on the activity being carried out instead of behaving automatically; (iv) *non-judging* of inner experience, which refers to the ability to take a non-evaluative stance toward thoughts and feelings; and (v) *non-reactivity* to inner experience. Items are rated on a Likert scale ranging between 1 (never or very rarely true) and 5 (very often or always true). The Spanish version of the FFMQ-15 has shown to be a reliable and a valid instrument in adult populations (Cronbach’s α ranged from 0.56 to 0.85). Confirmatory factor analyses showed the four-facet bifactor structure (mindfulness plus four specific facets, excluding observing) as the best-fitting model for the FFMQ-15. In the present study, Cronbach’s alpha values were as follows: *observing*, *α*=0.59; *describing*, *α*=0.81; *acting with awareness*, *α*=0.74; *non-judging*, *α*=0.82; and *non-reactivity*, *α*=0.57.

#### International Positive and Negative Affect Schedule Short Form

The international positive and negative affect schedule short form (I-PANAS-SF; Thompson, 2007) is a shortened form from the original 20-item PANAS that comprises 10 items to assess negative affect (NA) and positive affect (PA) traits. This instrument has been found to be psychometrically acceptable in a series of validation studies (*N*=1.789) when exploring cross-sample stability, internal reliability, temporal stability, cross-cultural factorial invariance, and convergent and criterion-related validity. The original instructions were modified to assess how the person had been feeling lately, and the Likert scale was type scoring modified from 0 (not at all) to 4 (very much). This Spanish adaptation is currently under validation. In the present study, the Cronbach’s alpha values were 0.82 for positive affect and 0.73 for negative affect.

### Data Analysis

Prior to analysis, missing data and patterns of acquiescent answers were observed. Descriptive statistics of the LuCiD items were subsequently examined, which included the mean, SD, skewness, and kurtosis. Second, to assess the construct validity of the scale, we tested the factorial structure proposed by the original version (Voss et al., 2013). To do so, we conducted a confirmatory factor analysis (CFA) to confirm the scale’s factorial structure in which the original 28-item version was tested: Factor 1: *Insight* (items 1, 3, 8, 9, 16, and 19), Factor 2: *Control* (items 4, 6, 10, 14, and 23), Factor 3: *Thought* (items 5, 12, and 22), Factor 4: *Realism* (items 7, 17, and 20), Factor 5: *Memory* (items 2, 13, 18, and 24), Factor 6: *Dissociation* (items 11, 15, and 21), Factor 7: *Negative emotion* (items 26 and 28), and Factor 8: *Positive emotion* (items 25 and 27). As in the study of Voss et al. (2013), we conducted CFA using the *MPlus* program, version 6.1 (Muthén and Muthén, 2010). The CFA was based on polychoric correlations. The *weighted least square mean and variance adjusted* (WLSMV) estimator was used. Model fit was assessed using the following goodness-of-fit statistics: *χ*^2^ (chi-square), with the lowest values indicating a better fit; the *root mean square error of approximation* (RMSEA)≤0.06; the 90% CI for RMSEA<0.08 (Brown and Cudeck, 1993); the *comparative fit index* (CFI)>0.90; and the *Tucker–Lewis index* (TLI)>0.90 (Kline, 2011). Third, t reliability values were obtained through both internal consistency – Cronbach’s alpha – and test–retest reliability using Pearson correlations. Fourth, in order to test construct validity, we conducted Pearson correlations among all the factors from LuCiD. Finally, to provide evidence of concurrent validity, we conducted *Student’s t-test* mean comparisons to examine whether there were significant differences by sex and by meditators vs. non-meditators. Pearson correlations among the factors from the LuCiD and all the examined variables – age, frequency of meditation, and scores from the FFMQ and PANAS – also were performed.

## Results

### Descriptive Statistics of Items

Table 1 shows the descriptive statistics for the LuCiD items – means, SDs, skewness, and kurtosis. The highest mean scores were shown for items from *Realism*, while the lowest scores were shown for items from *Dissociation*. Skewness and kurtosis values were considered to follow a normal distribution when values ranged from −1 to +1 and from −1 to +2, respectively (Huck, 2000). Within the *Dissociation* factor, item 15 (“*while dreaming, I was not myself but a completely different person*”) yielded both skewness and kurtosis, with values close to or greater than 2 for skewness and kurtosis, respectively. This indicates a non-normal distribution of responses for this item, in which most of the responses fell on the 0–1 options. In addition, the corrected item–total correlation was below 0.40 (Nunnally, 1978; Nunnally and Bernstein, 1994) and the Cronbach’s alpha value for *Dissociation* would have improved if item 15 had been eliminated. For these reasons, we eliminated item 15. Items 14 and 16 showed a slight skewness (>1); however, their item–total correlation was close to or above 0.40, and reliability was practically unaffected by their elimination.

**Table 1:** Means, SDs, skewness, kurtosis, item–total corrected correlation, and Cronbach’s alpha if item deleted.

Factor	Items no.	*M*	*SD*	Skewness	Kurtosis	*rit*	*α-i*
Insight	1	2.22	1.93	0.18	−1.49	0.63	0.74
	3	1.78	1.71	0.48	−1.05	0.56	0.76
	8	1.58	1.72	0.70	−0.89	0.52	0.77
	9	1.78	1.73	0.61	−0.91	0.63	0.74
	16	1.29	1.62	1.05	−0.13	0.38	0.80
	19	1.44	1.66	0.86	−0.57	0.56	0.76
	Total						0.79
Control	4	1.50	1.68	0.81	−0.64	0.50	0.76
	6	1.90	2.03	0.48	−1.45	0.53	0.77
	10	1.73	1.76	0.61	−1.03	0.66	0.72
	14	1.15	1.63	1.26	0.27	0.58	0.75
	23	1.72	1.68	0.56	−0.98	0.56	0.75
	Total						0.79
Thought	5	2.23	1.80	0.09	−1.38	0.45	0.75
	12	2.46	1.85	−0.05	−1.42	0.59	0.59
	22	2.44	1.73	−0.05	−1.32	0.61	0.57
	Total						0.73
Realism	7	3.24	1.63	−0.61	−0.76	0.47	0.57
	17	2.70	1.71	−0.21	−1.19	0.57	0.43
	20	2.87	1.68	−0.31	−1.10	0.39	0.67
	Total						0.66
Memory	2	2.49	1.88	−0.04	−1.43	0.40	0.62
	13	1.35	1.62	0.97	−0.34	0.37	0.63
	18	1.79	1.75	0.50	−1.13	0.46	0.57
	24	1.54	1.69	0.73	−0.81	0.52	0.53
	Total						0.65
Dissociation	11	1.38	1.72	0.86	−0.73	0.60	0.45
	15^*^	0.78	1.30	1.73	2.27	0.35	0.76
	21	1.54	1.67	0.70	−0.84	0.59	0.48
	Total						0.69
Negative emotion	26	1.97	1.74	0.39	−1.16	0.62	_
	28	2.01	1.73	0.35	−1.17	0.62	_
	Total						0.77
Positive emotion	25	2.26	1.74	0.15	−1.25	0.72	_
	27	2.37	1.64	0.01	−1.14	0.72	_
	Total						0.83

M, means; SD, standard deviations; ri-t, item–total corrected correlations; and α-i Cronbach’s alpha if item deleted.

* Item deleted.

### Confirmatory Factor Analysis

Two models were tested: (1) the original factorial structure from Voss et al. (2013) in which item 15 was eliminated from the *Dissociation* factor, and (2) a single-factor model for comparison. As seen in Table 2, the goodness-of-fit indices were good for the eight-factor model, with all values above the cutoff. However, when testing the single-factor model, the model did not achieve a good fit, as all the goodness-of-fit indices were below the cutoff. Standardized factor loadings ranged from 0.53 (item 13) to 0.93 (item 28).

**Table 2:** Model fit indices.

	*χ*^2^	*df*	RMSEA	90% CI RMSEA	CFI	TLI
Eight-factor model	782.40	296	0.06	0.06–0.07	0.93	0.91
Single-factor model	2819.18	324	0.14	0.14–0.15	0.64	0.61

Table 3 shows correlations for the eight factors of LuCiD, where all factors were significantly correlated with each other, except for *Realism*, which was not correlated to *Insight*, *Control*, *Dissociation*, or *Negative emotion*. In addition, *Positive emotion* and *Negative emotion* were not correlated.

**Table 3:** Correlations matrix for the eight factors of Spanish version of the lucidity and consciousness in dreams scale (LuCiD).

		1	2	3	4	5	6	7	8
1.	Insight								
2.	Control	0.61^***^							
3.	Thought	0.45^***^	0.50^***^						
4.	Realism	0.00	−0.02	0.24^***^					
5.	Memory	0.55^***^	0.49^***^	0.60^***^	0.31^***^				
6.	Dissociation	0.39^***^	0.33^***^	0.40^***^	0.06	0.40^***^			
7.	Negative emotion	0.09	0.06	0.22^***^	0.09	0.24^***^	0.19^***^		
8.	Positive emotion	0.30^***^	0.43^***^	0.38^***^	0.23^***^	0.34^***^	0.18^***^	−0.10	
9.	Total score	0.78^***^	0.77^***^	0.76^***^	0.32^***^	0.80^***^	0.58^***^	0.29^***^	0.54^***^

*** *p*<0.001.

### Reliability

Adequate values of internal consistency were observed through Cronbach’s alpha values for each dimension, with the lowest Cronbach’s alpha values for *Memory* (.=0.65) and the highest for *Positive emotion* (*α*=0.83; Table 1).

Regarding test–retest reliability, Pearson correlations were significant at a *p*<0.001 level between each corresponding factor, i.e., *Insight* (*r*=0.62), *Control* (*r*=0.70), *Thought* (*r*=0.48), *Realism* (*r*=0.51), *Memory* (*r*=0.65), *Dissociation* (*r*=0.64), *Negative emotion* (*r*=0.62), and *Positive emotion* (*r*=0.44).

### Evidence of Validity: Concurrent Validity

#### Differences in Sex and Age

When comparing men and women, significant differences were only found for *Negative emotion* [*F*_(365)_=7.48, *p*<0.05], for which men reported lower scores (*M*=1.72, *SD*=1.37) than women (*M*=2.12, *SD*=1.63). With regard to age, we found significant and negative correlations between age and *Control* (*r*=−0.11 *p*<0.05), *Thought* (*r*=−0.17 *p*<0.01), *Memory* (*r*=−0.14 *p*<0.01), and *Positive emotion* (*r*=−0.16 *p*<0.01), indicating that older individuals reported lower scores on these factors than younger individuals.

#### Meditators vs. Non-meditators and Experience With Meditation

There were significant differences for *Insight* [*F*_(365)_=9.95, *p*<0.01] and *Dissociation* [*F*_(365)_=2.06, *p*<0.01]. In particular, those who had experience with meditation reported higher scores for *Insight* (*M*=1.91, *SD*=1.33) and *Dissociation* (*M*=3.53, *SD*=3.12) than non-meditators [(*M*=1.52, *SD*=1.11; *M*=2.50, *SD*=2.94), respectively]. Regarding whether the time they had dedicated to meditation was associated with LuCiD scores, the results showed that only *Control* (*r*=0.20, *p*<0.01) and *Dissociation* (*r*=0.15, *p*<0.05) were significantly correlated with greater length of meditation experience. For the percentage of time dedicated to each type of meditation practice, results showed a significantly positive correlation between open monitoring practice and *Insight* (*r*=0.20; *p*<0.05), *control* (*r*=0.27; *p*<0.01), and a significant negative correlation to *Realism* (*r*=−0.21; *p*<0.05). Percentage of Compassion and loving-kindness meditation was significantly correlated to *Negative emotion* (*r*=0.226; *p*<0.01).

#### Factors of LuCiD, FFMQ, and PANAS

Correlations between the factors of LuCiD, FFMQ, and I-PANAS-SF are shown in Table 4. The results indicated that most of the factors from LuCiD were significantly correlated with one of the FFMQ facets, in particular with *Observing*. Therefore, FFMQ-Observing was positively correlated with all LuCiD factors except *Realism* and *Positive emotion*. However, *realism* was significantly and negatively correlated with FFMQ-*Acting Aware*. When observing the correlations from the I-PANAS-SF and the LuCiD factors, the results showed that I-PANAS-SF-Positive Affect was negatively correlated with *Negative emotion*, while I-PANAS-SF-Negative Affect was positively correlated with *Negative emotion* and negatively with *Positive emotion*.

**Table 4:** Correlations matrix between the eight factors of LuCiD and all the examined variables.

	Observing	Describing	Awareness	Non-judging	Non-reactivity	FMMQ total	PA	NA
Insight	0.17^**^	0.10^*^	0.08	−0.23	0.33	0.11^*^	−0.00	0.00
Control	0.10^*^	0.04	0.00	−0.01	0.04	0.05	−0.02	0.01
Thought	0.18^***^	0.09	−0.04	−0.00	0.04	0.08	0.01	0.07
Realism	0.10	0.02	−0.13^*^	−0.01	0.03	0.00	−0.02	−0.04
Memory	0.10^*^	0.02	−0.08	−0.06	−0.10	−0.03	−0.08	0.07
Dissociation	0.17^**^	0.04	−0.02	0.05	0.02	0.09	−0.04	0.07
Negative emotion	0.10^*^	−0.05	−0.20^**^	−0.17^**^	−0.09	−0.12^*^	−0.16^**^	0.29^***^
Positive emotion	0.08	0.02	0.00	0.02	0.07	0.06	−0.02	−0.11^*^

FFMQ, five facets mindfulness questionnaire; PA, positive affect; and NA, negative affect. *p < 0.05; **p < 0.01; ***p < 0.001.

## Discussion

The main purpose of the present study was to validate a Spanish version of the LuCiD. Specifically, factorial structure was examined by testing an eight-factor model for reliability by both internal consistency/test-retest and construct/concurrent validity of the scale in a sample of the Spanish general population. Overall, the results showed that the Spanish version of LuCiD is a valid and reliable measure.

As expected in the first hypothesis, CFA confirmed the eight-factor model originally proposed and validated by Voss et al. (2013), by supporting the eight factors of lucidity and consciousness in dreams: *Insight*, *Control*, *Thought*, *Realism*, *Memory*, *Dissociation*, *Negative emotion*, and *Positive emotion*. The original eight-factor model tested showed goodness of fit in contrast to a single-factor model in which items only belong to one global factor. These results are in line with those found by Voss et al. (2013), who identified and recommended eight factors involved in dream consciousness. Moreover, our study also adds new data by comparing the 8-factor structure vs. single-factor model global factor, where the use of one global factor to explain lucidity and consciousness in dreams was not supported nor recommended. The original 28 items showed adequate factor loadings as expected, similar to Voss et al. (2013). Nevertheless, item 15 was deleted from the *dissociation* factor as it performed poorly, as indicated in Results section. In our study, the item 15 mean was 0.78, and most of the participants answered either 0 or 1. This was the lowest score in comparison with the rest of the items, which may indicate low discriminative power. These results are congruent with Voss et al. (2013) where the mean score for item 15 was low (0.40). In addition to statistical reasons, we consider some conceptual reasons to delete it: while the item 11 (“*while dreaming, I saw myself from outside”*) and item 21 (“*watched the dream from the outside, as if on a screen*”) refer to “see oneself from outside,” item 15 refers to a different person (“*while dreaming I was not myself but a completely different person*”). This may explain the low factor loadings of this item in the *dissociation* factor.

Eight factors from the LuCiD scale presented adequate internal consistency, ranging from 0.65 to 0.83, which can be regarded as acceptable (0.60–0.70) and very good (0.80 or greater; Ursachi et al., 2015). These results are comparable to the original validation, where most factors showed good reliability. While Voss et al. (2013) found reliability levels slightly lower than desired for *memory* (0.66) and *negative emotion* (0.68), and too low for *dissociation* (0.56), we found the lowest levels for *realism* (0.66), *memory* (0.65), and *dissociation* (0.69). Differences in the *dissociation* factor may be due to the elimination of item 15. It is important to mention that although these were the lowest reliability values, they can still be considered to have good and adequate internal consistency. Moreover, because of their high discriminative power with regard to lucidity and consciousness in dreams, they can therefore be considered valid (Moosbrugger and Kelava, 2011; Voss et al., 2013). Furthermore, we also found significant test–retest reliability for each factor after a period of 10–15days.

Construct validity was supported by significant correlations between the eight factors of LuCiD except for *Realism*, which was not correlated with *Insight*, *Control*, *Dissociation*, or *Negative emotion*. In addition, *Positive emotion* and *Negative emotion* were not correlated. In our study, the most noticeable correlations (*r*>0.50) were found between *Insight* and *Control*, *Memory* and *Thought*, *Insight* and *Memory*, and *Thought* and *Control*. These findings are congruent with those found by Voss et al. (2013), who highlighted an interesting relationship between *Insight*, *Control* and *Thought* as having the leading role in LDs together with *Positive emotion* and *Dissociation*. In this regard, *Control* and *Insight* are considered defining characteristics of LDs, although control seems to be subordinate to lucid insight because it is normally only experienced in the presence of lucid insight (Voss et al., 2012, 2013). For the *Memory* and *Thought* association, as Voss et al. (2013) concluded, both constructs are related and difficult to separate. Findings regarding *Realism* are in line with those found in the original validation, where this factor was not significant to differentiate lucid and non-lucid dreams, suggesting that the events in lucid and non-lucid dreams are equally realistic, or equally lacking in bizarre features. Voss et al. (2013, p.19) concluded that “lucidity involves the cognitive realization that you are currently dreaming or the ability to conceptualize ongoing experience as a dream, not necessarily experiencing your dreams as unreal or as a merely virtual reality.”

It is also important to note that there were several differences in the LuCiD scores observed regarding the scales related to dream lucidity (i.e., *Insight* and *Control*) compared to those obtained by Voss et al. (2013), whose scores were lower than those of the present study, suggesting higher prevalence of lucid dreaming in the present sample. However, these findings must be interpreted with caution due to differences in procedures and data collection, as mentioned in “Limitations and Future Research Directions” section.

For the second hypothesis, evidence for validity was tested. Our findings showed a similar tendency for LuCiD scores between men and women except for *Negative emotion*, which indicated lower scores for men. These data are in line with Voss et al. (2013), who found the same tendency, including differences in sex for other factors (*Control* and *Thought*), but no differences regarding non-lucid dreams. The authors reported such differences but refrained from interpreting them because their sample was not representative. A possible explanation may be related to those data showing gender differences in emotional response, indicating higher emotional expressivity, particularly for negative emotions expressed by women (Deng et al., 2016). A meta-analysis exploring gender differences in dream recall found robust findings of gender differences that are affected by age showing different effect sizes and greater effects for adolescents and adults (Schredl and Reinhard, 2008). With regard to the role of age, older participants reported significantly lower scores for *Control*, *Thought*, *Memory*, and *Positive emotion*. Further research is needed to interpret these findings and explore the role of age in LDs and the possible influence of other factors (e.g., personality factors) that have not been dealt with in this study.

When comparing participants with and without meditation experience and whether they practiced meditation (meditators vs. non-meditators), the results revealed significant differences for *Insight* and *Dissociation*, indicating higher scores for meditators. Moreover, length of meditation experience was significantly associated with *Control* and *Dissociation* (i.e., higher scores for more meditation experience). Our findings showed the role of meditation practice in promoting key factors of lucid dreaming such as insight, control, and dissociation. Our findings are in line with studies indicating the influence of meditation practice on lucid dreaming (e.g., Gasca and García-Campayo, 2017), supporting that lucid dreaming occurs more frequently in long-term meditators than in meditation-naïve individuals (Baird et al., 2019). An exploratory analysis suggested significant correlations between the percentage of the time dedicated to some type of meditation practice and certain LuCiD factors. As mentioned in the results, a positive correlation was found for open monitoring practice with *Insight* and *Control*, and a negative correlation with *Realism*. In addition, the percentage of Compassion and Loving-Kindness meditation was significantly correlated to *Negative emotion*. These results may indicate a differential effect of different meditation practices (given their cognitive mechanism of action) and the promotion of lucid dreams differentially affecting LuCiD factors. Nevertheless, these data should be interpreted with caution, and rigorous and well-designed studies to further investigate this issue should be addressed.

For dispositional mindfulness, the most noticeable result was that the *Observing* facet was associated with six LuCiD factors (all except *Realism* and *Positive emotion*), which may suggest the influence of the “individual’s capacity to pay attention to internal and external experiences such as sensations, thoughts, and emotions” (Baer et al., 2006, 2008) in lucid dreaming. These results are congruent with those reported by Baird et al. (2019) in which LD frequency in long-term meditators was associated with the observational and decentering facets of trait mindfulness. It is important to mention that the observing facet of mindfulness has shown a controversial feature in mindfulness research, which points out that observing is one of the facets most related to and influenced by meditative practice (e.g., Lilja et al., 2013; Soler et al., 2014; Campos et al., 2019). Furthermore, total scores from the mindfulness questionnaire were also positively correlated with *Insight* and negatively correlated with *Negative emotion*. This is a relevant finding, given that insight has been highlighted as one of the key factors for LDs (e.g., Voss et al., 2012, 2013). Therefore, according to Stumbrys et al. (2015), our finding supports an existing relationship between lucidity in dreams and mindfulness during wakefulness, yet it remains unclear whether the relationship is influenced by actual meditation practice or whether it reflects some natural predispositions. People practicing mindfulness or showing higher dispositional mindfulness may have greater capacity to pay attention to the present, increasing their awareness of the ongoing experience, which seems to translate into a greater consciousness in dreams promoting lucid dreaming. Moreover, dispositional mindfulness may also promote dream control as supported by Stumbrys and Erlacher (2017), who found that higher dispositional mindfulness and younger age are predictors of dream control. Taken together, these findings highlight the key role of trait mindfulness on LDs. Finally, positive and negative affects were associated with the LuCiD factors *Positive emotion* and *Negative emotion*. These findings may suggest the influence that dispositional positive and negative trait affects have on lucid dreaming, particularly related to both the *Negative emotion* and *Positive emotion* factors, which may be particularly important to consider for LD induction or therapeutic settings such as LDT. In addition, it is worthy of note that negative affect in the wakefulness state had a stronger relationship with both positive and negative emotions in dreams in comparison with positive affect. These finding may be related to the threat simulation theory of dreams (TST; Revonsuo, 2000; Valli and Revonsuo, 2009), which states that dream consciousness is essentially an ancient biological defense mechanism, evolutionarily selected for its capacity to repeatedly simulate threatening events (Valli et al., 2005).

### Limitations and Future Research Directions

Despite the encouraging results of this study, several limitations and methodological issues should be mentioned. The main limitations are related to the sample and data collection. On the one hand, participants were recruited over the Internet *via* an online survey, in contrast to Voss et al. (2013), who collected most of the data in a laboratory setting; this could have resulted in selection bias regarding the overrepresentation of specific groups of people (i.e., people interested in LDs, mindfulness, or meditation) and affected the scores obtained in the present study. Moreover, although participants were instructed to answer the LuCiD questionnaire by selecting a recent dream, the period between the specific dream and their responses was not controlled, in contrast with Voss et al. (2013), who measured a specific very recent dream experience – either a dream collected immediately after awakening from REM sleep in a sleep laboratory or a dream that occurred less than 6h previously. Participants’ responses were considered a state, as in the original LuCiD questionnaire, rather than a trait because we assumed that responses were provided related to a recent and specific dream and not measuring a tendency or trait. However, data in this regard were not recorded and should be further explored in future studies. This may influence the results of the present study because a longer period would have an impact on the recollection of a dream experience. Retest reliability may have also been influenced as the correlations would be expected to be much stronger in those participants reporting dreams after a longer period – to measure the trait rather than the state.

These methodological and construct issues should be considered when interpreting data from this study, and future studies with the Spanish validation should control for them.

Furthermore, the final sample represents a highly educated population that might not be representative of the general population, which may compromise the generalizability of the findings and show different psychometric properties and factor structures, suggesting a relevant research target for future studies. Nevertheless, our sample is similar to that tested by Voss et al. (2013), where the study was mainly advertised among university students. In addition, given that final sample was composed of a Spanish population, further cross-cultural validation is required to confirm that our findings are equivalent in other Spanish-speaking cultures (e.g., populations from Latin America; e.g., Huang and Wong, 2014).

With regard to other methodological issues, given the correlational nature and cross-sectional design of this study, causal inferences were not possible. Moreover, as in any study using self-report measures, the results might be influenced by participants’ acquiescence and need for social desirability.

Finally, data regarding whether participants had lucid dreams were not collected, which did not allow us to compare lucid and non-lucid dreamers, as done by Voss et al. (2013). Participants were asked to select any recent dream but not a recent lucid dream specifically, which may affect the present data. Future studies using the Spanish version of LuCiD should include this variable, together with dreams report, to explore the ability of the scale to differentiate between lucid and non-lucid dreams, as well as to study further differences observed in mean scores for some items compared to the original scale, as mentioned above.

For future research directions, it would be interesting to further test the psychometric properties of the validated Spanish LuCiD among specific samples (e.g., less highly educated, psychiatric samples, Latin American population and people with high dispositional mindfulness). As suggested by Stumbrys et al. (2015), another line of research is to promote a deeper understanding of the relationship between meditation practice and LDs by examining the roles of the different types of meditation practiced, to investigate personality variables that might influence this relationship and further explore how different facets of mindfulness and lucidity interrelate. Moreover, more studies on the effects of mindfulness (e.g., trait vs. state) and the association between mindfulness-based intervention and LD induction are still needed.

To conclude, this study provides a validated measure of lucidity and consciousness in dreams in Spanish population. To the best of our knowledge, no validation has been previously performed of LuCiD in any other language, apart from the original English version. This study adds data to the lucid dreaming research supporting the psychometric properties of the LuCiD and provides a valid and reliable self-report questionnaire to assess lucidity and consciousness in dreams for the Spanish general population. Moreover, our findings suggest relevant associations between lucid dreaming, meditation experience, mindfulness trait, and positive and negative affect.

## Data Availability Statement

The raw data supporting the conclusions of this article will be made available by the authors, without undue reservation.

## Ethics Statement

All procedures performed in studies involving human participants were in accordance with the ethical standards of the Institutional and/or National Research Committee and with the 1964 Declaration of Helsinki and its later amendments or comparable ethical standards. This study was approved by the Clinical Research Ethics Committee of Aragón (Comité Ético de Investigación Clínica de Aragón, CEICA) which is part of the Health Research Institute of Aragon (Instituto de Investigación Sanitaria de Aragón, IIS Aragón), Zaragoza, Spain. Informed consent was obtained online from all individual participants included in the study.

## Author Contributions

DC drafted the first version of the manuscript with important contributions from JG-C. NM performed the statistical analysis and wrote the results sections with contributions from DC. JG-C designed and planned the study, assisted the data analyses, and collaborated in the writing and editing of the draft and the final manuscript. MM-A and DC conducted the online recruitment and the data set accuracy. PH-M, MP-G, and SG contributed to the literature searches and collaborated on the writing and editing of the final manuscript. All authors contributed to the article and approved the submitted version.

## Funding

This study was partially supported by the Master in Mindfulness Programme and the Chair of Contemplative Sciences, University of Zaragoza (Zaragoza, Spain), and the Instituto de Investigación Sanitaria Aragón (IIS Aragón; Zaragoza, Spain). The funding source had no influence on the design of the study, data collection and analysis, or writing of the manuscript.

## Conflict of Interest

The authors declare that the research was conducted in the absence of any commercial or financial relationships that could be construed as a potential conflict of interest.

## Publisher’s Note

All claims expressed in this article are solely those of the authors and do not necessarily represent those of their affiliated organizations, or those of the publisher, the editors and the reviewers. Any product that may be evaluated in this article, or claim that may be made by its manufacturer, is not guaranteed or endorsed by the publisher.

## Footnotes

^1^ https://es.surveymonkey.com/

^2^ https://soñarlucido.com/validacion-lucid/
