# Supplementary material for: Validation of the Spanish Version of the Lucidity and Consciousness in Dreams Scale
Source: Front Psychol. 2021 Oct 21;12:742438. doi: 10.3389/fpsyg.2021.742438 (PMC8566340; doi:10.3389/fpsyg.2021.742438)
Supplement: Supplementary file 1 [file Table_1.DOCX]

**Supplementary Material**

*Spanish version of the Lucidity and Consciousness in Dreams (LuCiD) scale*

A continuación, selecciona un sueño reciente que hayas tenido y contesta a las siguientes preguntas relativas al mismo:

| **0** | | **1** | **2** | **3** | **4** | **5** |
| --- | --- | --- | --- | --- | --- | --- |
| Totalmente en desacuerdo |  |  |  |  | Totalmente de acuerdo |  |

| 1. | Mientras soñaba, me di cuenta de que las cosas que estaba experimentando en el sueño no eran reales. | 0 | 1 | 2 | 3 | 4 | 5 |
| --- | --- | --- | --- | --- | --- | --- | --- |
| 2. | Mientras soñaba era capaz de recordar mi intención de hacer ciertas cosas en el sueño. | 0 | 1 | 2 | 3 | 4 | 5 |
| 3. | Mientras soñaba, me di cuenta de que el self que experimentaba durante el sueño era distinto del self de vigilia. | 0 | 1 | 2 | 3 | 4 | 5 |
| 4. | En el sueño, era capaz de controlar o manipular a otros personajes del sueño de una manera que sería imposible en vigilia. | 0 | 1 | 2 | 3 | 4 | 5 |
| 5. | Mientras soñaba, pensaba en otros personajes del sueño. | 0 | 1 | 2 | 3 | 4 | 5 |
| 6. | Mientras soñaba, era capaz de realizar con éxito acciones sobrenaturales (como volar, o traspasar muros). | 0 | 1 | 2 | 3 | 4 | 5 |
| 7. | Las emociones que experimentaba en el sueño eran exactamente las mismas que podría experimentar en las mismas situaciones durante la vigilia. | 0 | 1 | 2 | 3 | 4 | 5 |
| 8. | Mientras soñaba, me daba cuenta del hecho de que el cuerpo que experimentaba en el sueño no se correspondía con mi cuerpo de vigilia que estaba dormido. | 0 | 1 | 2 | 3 | 4 | 5 |
| 9. | Estaba muy seguro de que las cosas que estaba experimentando en el sueño no tenían consecuencias en el mundo real. | 0 | 1 | 2 | 3 | 4 | 5 |
| 10. | Mientras soñaba, era capaz de controlar o cambiar el escenario del sueño de una manera que sería imposible en vigilia. | 0 | 1 | 2 | 3 | 4 | 5 |
| 11. | Mientras soñaba, me observé a mí mismo desde el exterior. | 0 | 1 | 2 | 3 | 4 | 5 |
| 12. | Mientras soñaba, pensé sobre mis propias acciones. | 0 | 1 | 2 | 3 | 4 | 5 |
| 13. | Mientras soñaba, tenía la sensación de que había olvidado algo importante. | 0 | 1 | 2 | 3 | 4 | 5 |
| 14. | Mientras soñaba, era capaz de cambiar o mover objetos (no personas) de una manera que sería imposible en vigilia. | 0 | 1 | 2 | 3 | 4 | 5 |
| 15. | Mientras soñaba*,* me daba cuenta de que no era yo mismo sino una persona completamente distinta. | 0 | 1 | 2 | 3 | 4 | 5 |
| 16. | Mientras soñaba, me preguntaba a mí mismo frecuentemente si era un sueño. | 0 | 1 | 2 | 3 | 4 | 5 |
| 17. | Los pensamientos que tenía durante el sueño eran exactamente los mismos que hubiera tenido en una situación similar en vigilia. | 0 | 1 | 2 | 3 | 4 | 5 |

| 18. | | | Mientras soñaba, tuve la sensación de que podía recordar mi vida de vigilia. | 0 | 1 | | 2 | | 3 | | | 4 | | 5 | |
| --- | --- | --- | --- | --- | --- | --- | --- | --- | --- | --- | --- | --- | --- | --- | --- |
| 19. | | | Mientras soñaba, me daba cuenta del hecho de que otros personajes del sueño no eran reales. | 0 | 1 | | 2 | | 3 | | | 4 | | 5 | |
| 20. | | | Muchas cosas que ocurrieron en el sueño podrían haber ocurrido durante la vigilia. | 0 | 1 | | 2 | | 3 | | | 4 | | 5 | |
| 21. | | | Pude observar el sueño desde el exterior, como si lo viera en una pantalla. | 0 | 1 | | 2 | | 3 | | | 4 | | 5 | |
| 22. | | | Mientras soñaba, en muchas ocasiones pensé sobre lo que estaba experimentando. | 0 | 1 | | 2 | | 3 | | | 4 | | 5 | |
| 23. | | | Era capaz de influir en el desarrollo de la historia del sueño a voluntad. | 0 | 1 | | 2 | | 3 | | | 4 | | 5 | |
| 24. | | | Mientras soñaba, fui capaz de recordar ciertos planes de futuro. | 0 | 1 | | 2 | | 3 | | | 4 | | 5 | |
| 25. | | | Mientras soñaba me sentí eufórico/optimistas. | 0 | 1 | | 2 | | 3 | | | 4 | | 5 | |
| 26. | | | Mientras soñaba, tuve fuertes sentimientos negativos. | 0 | 1 | | 2 | | 3 | | | 4 | | 5 | |
| 27. | Mientras soñaba, tuve fuertes sentimientos positivos. | | 0 | | 1 | | 2 | | 3 | 4 | | 5 | |  |  |
| 28. | Mientras soñaba, me sentí muy ansioso. | | 0 | | 1 | | 2 | | 3 | 4 | | 5 | |  |  |

| **0** | | **1** | **2** | **3** | **4** | **5** |
| --- | --- | --- | --- | --- | --- | --- |
| Totalmente en desacuerdo |  |  |  |  | Totalmente de acuerdo |  |
